# Recent Advances in Halogen-Free Flame Retardants for Polyolefin Cable Sheath Materials

**DOI:** 10.3390/polym14142876

**Published:** 2022-07-15

**Authors:** Yan Li, Leijie Qi, Yifan Liu, Junjie Qiao, Maotao Wang, Xinyue Liu, Shasha Li

**Affiliations:** 1 School of Electrical and Electronic Engineering, North China Electric Power University, Baoding 071003, China; 220192213283@ncepu.edu.cn (L.Q.); 220192213307@ncepu.edu.cn (Y.L.); junjie_qiao@ncepu.edu.cn (J.Q.); 220212213259@ncepu.edu.cn (M.W.); 220212213013@ncepu.edu.cn (X.L.); 2 State Grid Hebei Baoding Electric Power Company Limited, Baoding 071051, China; chengfengpolanglyf@163.com

**Keywords:** cable sheath material, polyolefin, halogen-free flame retardant, mechanical properties

## Abstract

With the continuous advancements of urbanization, the demand for power cables is increasing to replace overhead lines for energy transmission and distribution. Due to undesirable scenarios, e.g., the short circuit or poor contact, the cables can cause fire. The cable sheath has a significant effect on fire expansion. Thus, it is of great significance to carry out research on flame-retardant modification for cable sheath material to prevent fire accidents. With the continuous environmental concern, polyolefin (PO) is expected to gradually replace polyvinyl chloride (PVC) for cable sheath material. Moreover, the halogen-free flame retardants (FRs), which are the focus of this paper, will replace the ones with halogen gradually. The halogen-free FRs used in PO cable sheath material can be divided into inorganic flame retardant, organic flame retardant, and intumescent flame retardant (IFR). However, most FRs will cause severe damage to the mechanical properties of the PO cable sheath material, mainly reflected in the elongation at break and tensile strength. Therefore, the cooperative modification of PO materials for flame retardancy and mechanical properties has become a research hotspot. For this review, about 240 works from the literature related to FRs used in PO materials were investigated. It is shown that the simultaneous improvement for flame retardancy and mechanical properties mainly focuses on surface treatment technology, nanotechnology, and the cooperative effect of multiple FRs. The principle is mainly to improve the compatibility of FRs with PO polymers and/or increase the efficiency of FRs.

## 1. Introduction

**Figure 1:**
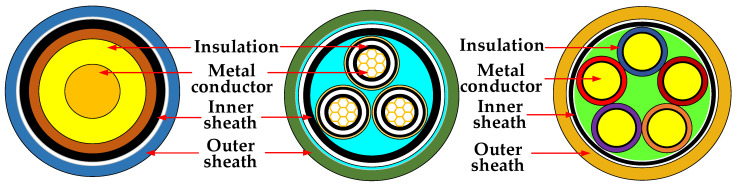
Schematic diagram of three typical cable structures.

Power cables are important equipment for energy transmission and are closely related to system security [1]. Due to complex working environment, different operating conditions, and flammable insulating materials, fire accidents of power cables often occur. The survey found that 50% of electrical fires are caused by burning cables [2]. Once a fire occurs, it will not only lead to the interruption of power transmission and cause major economic losses, but also affect the health and safety of people [3]. Three typical cables are listed in Figure 1. The polymer composite materials in the cable structure are mainly sheath and insulation. Thus, the cable sheath has a significant effect on fire expansion. Thus, it is beneficial to improve the flame retardancy of the cable sheath material. Meanwhile, the sheath material should maintain certain mechanical strength to meet relevant standard, e.g., the European standard EN 50264 for locomotives cables. As shown in Table 1, flame-retardant cable materials need to meet the indicators in terms of flame-retardant and mechanical properties.

At present, the materials used for cable sheath are mostly organic polymers, including PVC, polyethylene (PE), polypropylene (PP), ethylene vinyl acetate (EVA), etc., of which PE includes high-density polyethylene (HDPE), low-density polyethylene (LDPE), and linear low-density polyethylene (LLDPE) [4,5]. PVC is now widely used as sheath material because of its good electrical insulation properties, mechanical performance, and low price [6]. However, PVC generates highly dense smoke and releases toxic gases during burning. With the enhancement of environmental awareness, countries around the world actively advocate for the use of PO, which is more environmentally friendly, instead of PVC as cable material. However, since the molecular chain of the PO matrix is composed of two elements, i.e., C and H, its limiting oxygen index (LOI) is only about 18%, and the char-formation rate is relatively low with the production of a large volume of molten droplets during combustion [7]. Thus, FRs should be added into PO to improve its flame retardancy. Generally, halogen-free FRs are preferred due to their excellent flame-retardant property and environmental friendliness, while the polarity difference between most FRs and PO matrix may lead to the sacrifice of mechanical performance. Balancing the flame retardancy and mechanical parameters is crucial for new material development.

**Table 1:** Index of halogen-free flame-retardant cable sheath material [8].

Performance	Unit	Index
LOI	%	≥30
Tensile strength	MPa	≥10
Elongation at break	%	≥150

## 2. Flame Retardant Mode of Action

**Figure 2:**
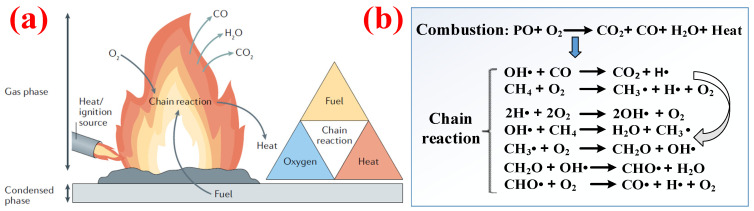
(**a**) Mode of action for combustion and the fire triangle. (**b**) Chain reactions occurring during PO combustion. Reprinted from Reference [9] with permission.

It is necessary to know about the interaction between the PO polymer and the ignition source before preparing an effective flame retardant. Figure 2a illustrates the combustion mode of action of combustible materials in air and the four key factors that initiate combustion: combustible material, oxygen, heat, and a chain reaction [9,10]. Zhao et al. [11] proposed that the combustion behavior of PO polymers can be roughly divided into five stages, namely heating, degradation, decomposition, ignition, and combustion, of which the decomposition stage will generate a large number of combustible gases (such as H_2_, CH_4_, CO, etc.) and thus intensify the combustion. Then a series of chain reactions occur, as shown in Figure 2b, and the products become combustible materials in combustion stage.

The corresponding basic theories of flame inhibition mode of action can be summarized as follows [11]:

(i) Covering effect: When heated, some FRs can form a non-flammable protective layer, thereby blocking the two elements, i.e., oxygen and heat, necessary for combustion.
(ii) Dilution effect: Some FRs can release incombustible gases, such as CO_2_ and water vapor, when heated, and this can reduce the oxygen concentration around the polymers, thereby inhibiting the combustion process.
(iii) Endothermic effect: Some FRs can undergo a decomposition reaction to absorb a large amount of heat, leading to a cooling effect on the polymer.
(iv) Inhibition effect: The thermal decomposition of some FRs will generate a large number of radicals, which can combine with the reactive radicals released by the polymer matrix to interrupt the process of the chain reaction.

Therefore, the action of FRs is mainly to interfere with the five stages of the combustion process according to these four modes of action, so as to reduce the spread of fire. This paper summarizes the research progress related to the halogen-free FRs, i.e., inorganic FRs, organic FRs, and IFR, which are used to cooperatively improve the flame-retardant properties and mechanical performance of PO matrix in recent years.

## 3. Inorganic Flame Retardants

### 3.1. Metal Hydroxide

**Figure 3:**
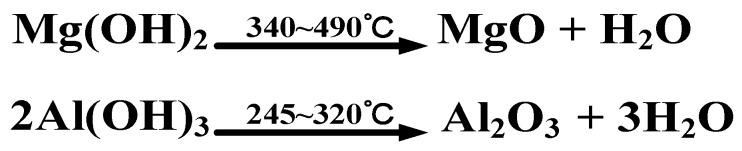
The decomposition reaction under high-temperature conditions of MH and ATH.

As FRs for PO materials, aluminum hydroxide (ATH) and magnesium hydroxide (MH) share the similar flame-retardant principle. The reaction formula is shown in Figure 3. Under high-temperature conditions, MH and ATH will produce water, which can absorb heat through vaporization to cool the material. Moreover, the percentage of oxygen in the air can be reduced due to the production of water vapor, thus achieving the effect of inhibiting or delaying combustion. At the same time, the products of MH and ATH after combustion, i.e., MgO and Al_2_O_3_, have high specific surface energy and strong adsorption capacity. They can cover the outer surface of the PO polymer, thus forming a protective barrier to suppress smoke and slow the spread of fire [12].

However, MH or ATH often require a large number of addition (50–60 wt.%) to achieve good flame-retardant properties. Furthermore, the polarity difference between hydroxide FRs and PO leads to poor compatibility, which seriously reduces the mechanical properties of the composites, such as elongation at break and tensile strength [13,14].

**Figure 4:**
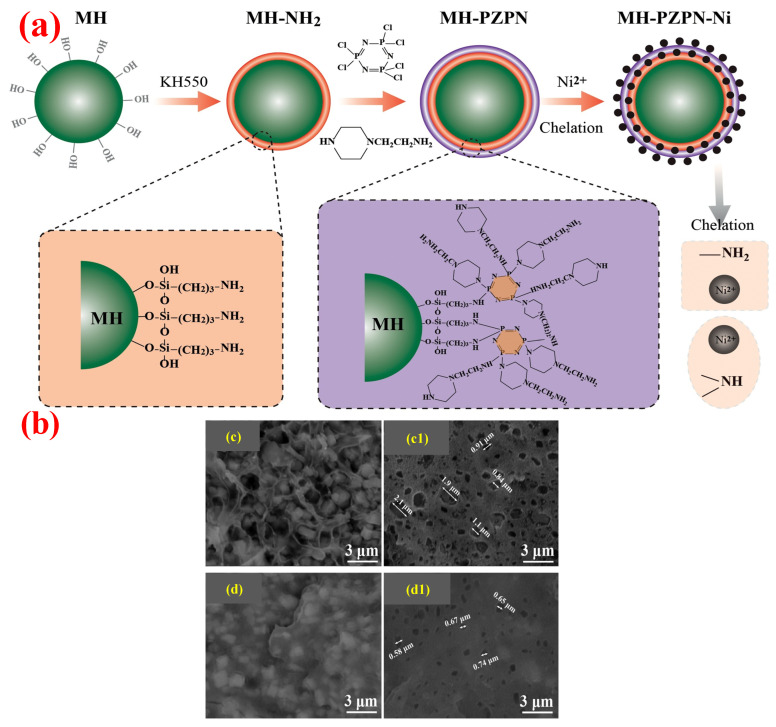
(**a**) Schematic of the synthesis of MH-PZPN-Ni. (**b**) SEM pictures of cryofractured sections: EVA/MH (**c**), EVA/MH-PZPN-Ni (**d**), and etched by hydrochloric acid (**c1**,**d1**). Reprinted from Reference [20] with permission.

To improve the flame retardancy and mechanical properties of PO composites, researchers have proposed the method of surface modification for MH/ATH, which can change the surface properties of the metal hydroxide to improve its interfacial adhesion with PO polymers [15,16]. Silane coupling agents, as a common surface modifier, have shown significant cooperative improvement in the flame retardancy and mechanical properties of PO composite systems [17,18]. Yang et al. [19] added γ-(2, 3-epoxypropoxy) propytrimethoxysilane (KH-560) to LLDPE/MH as a surface modifier for MH and concluded that the composite had the best cooperative properties when 3–7 wt.% KH-560 was added. On the one hand, KH-560 can improve the dispersion of MH in the PO matrix; on the other hand, the increase of KH-560 content leads to the larger particle size of MH in the system, and this is not conducive to improvement. Therefore, it is necessary to select the appropriate range of KH-560 content to obtain satisfactory composite properties. Meng et al. [20] synthesized a novel MH-polyphosphazene-Ni^2+^ (MH-PZPN-Ni) with core–shell structure by effectively grafting γ-aminopropyl triethoxysilane (KH-550), hexachlorocyclotriphosphazene (HCCP), 1-(2-Aminoethyl) piperazine (AEP), and Ni^2+^ on the surface of MH through the strategy of layer-by-layer assembly and method of Ni^2+^ chelation. The synthesis principle is shown in Figure 4a. They found that 60 wt.% MH-PZPN-Ni added into the EVA matrix could lead to the improvement of the obtained composites in both mechanical performance and flame retardancy, with the tensile strength and elongation at break of 11.4 MPa and 107.2%, respectively. Moreover, it achieved an LOI of 30.4% and vertical combustion test (UL-94) of V-0 rating. Meanwhile, compared with EVA/MH, pores presented on the surface of the EVA/MH-PZPN-Ni composites were smaller and fewer, as shown in Figure 4b, thus indicating that the compatibility between MH-PZPN-Ni and EVA matrix had been improved.

**Figure 5:**
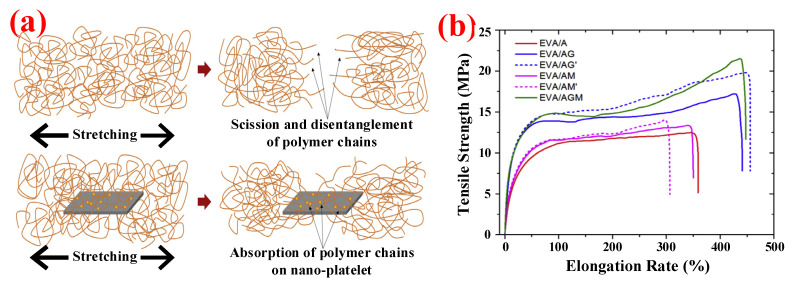
(**a**) Schematic diagram of the mode of action of GnPs. (**b**) Stress–strain diagram of EVA composites: (EVA, 60 wt.%; A, 40 wt.% ATH; AG, 38 wt.% ATH + 2 wt.% GnPs; AG’, 36 wt.% ATH + 4 wt.% GnPs; AM, 38 wt.% ATH + 2 wt.% MoS_2_; AM’, 36 wt.% ATH + 4 wt.% MoS_2_; and AGM, 36 wt.% ATH + 2 wt.% GnPs + 2 wt.% MoS_2_). Reprinted from Reference [26] with permission.

In addition, researchers have found that nano-hydroxide obtained by ultra-fabrication shows the characteristics of small volume size, low filling amount, and large specific surface area. Moreover, nanoscale particles are easier to disperse uniformly in the PO matrix; thus, they have less negative impact on the mechanical performance of the polymeric material when used as FRs [21]. Wang et al. [22] prepared PP composites with 50 wt.% nano-MH via the process of melt extrusion. The results showed that this method not only increased the value of LOI (from 19.3% to 29.1%), but also the sample of PP/MH passed the UL-94 test with a V-0 rating. Moreover, the nanoparticles can be dispersed well in PP polymer, which has a certain enhancement effect on the elongation at break and tensile strength of PP/MH composites. To obtain the cable materials with better mechanical properties, Liu et al. [23] synthesized MH nanoparticles grafted with 9,10-dihydro-9-oxa-10-phosphaphenanthrene-10-oxide (DOPO) via vinyl silane coupling agents WD70 and incorporated 51.32 wt.% of them into the EVA matrix by using the melt-blending method. The elongation at break and the LOI can reach 209% and 29.8%, respectively. Moreover, researchers have found that nano additives can be the flame-retardant adjuvant of metal hydroxide, which had a great effect on improving the flame retardancy and mechanical properties of PO materials with a low addition amount [24]. Yen et al. [25] used nanoclay as an adjuvant for EVA/48 wt.% MH composites. Due to the compact structure of the silicate layer formed by heated nanoclay, the thermal insulation effect of the metal oxide layer can be further enhanced; the LOI of EVA/48 wt.% MH polymers with 2 wt.% nanoclay reached 34.5%. Compared with sample test without nanoclay, an increase of about 25% LOI was observed, and the V-0 rating was maintained. Meanwhile, the elongation at break and tensile strength of the composites increased from 375% and 8.5 MPa to 396% and 9.4 MPa, respectively. Guo et al. [26] improved the flame retardancy and mechanical properties of EVA/40 wt.% ATH composites by adding 2 wt.% graphene nano-platelets (GnPs) and 2 wt.% MoS_2_. They prepared EVA/ATH and EVA/ATH/GnPs/MoS_2_ samples through the method of melt blending, in which the EVA/ATH composite was recorded an LOI of 26% and a V-2 rating in UL-94 test. The elongation at break and tensile strength were 359% and 12.6 MPa. After the introduction of GnPs and MoS_2_, the LOI of the composite was 29.5%, and the UL-94 test reached V-0 rating. The elongation at break and tensile strength were 448% and 21.5 MPa, respectively. Nanoplatelets appeared to increase resistance to deformation and improve the modulus at break as proposed in Figure 5a. Meanwhile, Figure 5b shows the effect of GnPs or MoS_2_ on the mechanical performance of EVA/ATH composites. The test results showed that GNPs had an adsorption effect on the polymer molecular chain. When the polymer is under tension, this adsorption can prevent the breaking of the molecular chain, thereby enhancing the mechanical properties of the polymer.

### 3.2. Inorganic Phosphorus

**Figure 6:**
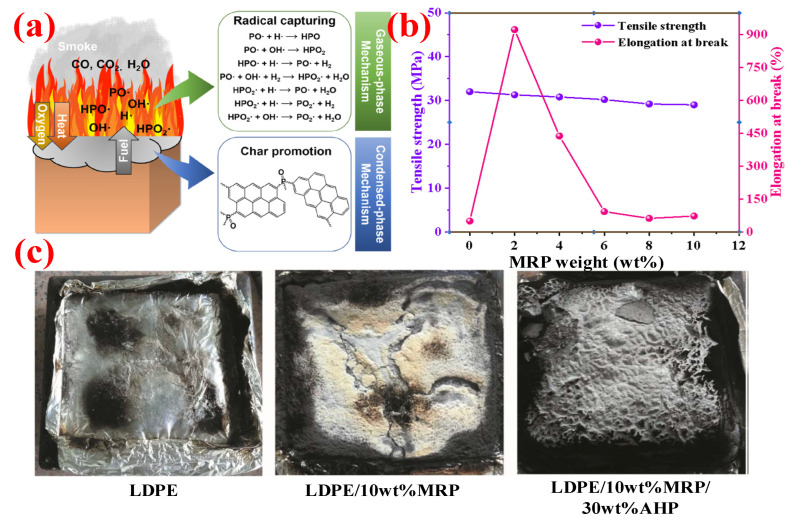
(**a**) The mode of action of phosphorus FRs. Reprinted from Reference [42] with permission. (**b**) Effect of MRP addition on mechanical properties of PP composites. (**c**) Pictures of the residual charcoal. Reprinted from Reference [41] with permission.

As one of the optimal alternatives for halogen-containing FRs [27], phosphorous-based FRs can be divided into organic phosphorus FRs and inorganic phosphorus FRs. The latter mainly include red phosphorus (RP) [28], ammonium polyphosphate (APP) [29], and phosphate (such as ammonium phosphate, ammonium dihydrogen phosphate, diammonium hydrogen phosphate, etc.) [30]. Generally speaking, there are two flame-retardant approaches for phosphorus-based FRs, namely the gas phase and condensed phase (shown in Figure 6a). In the gas phase, the PO matrix will produce radicals such as H·, HO·, and CH_3_· at high temperatures, while RP or APP generates PO· and PO_2_·. These radicals will combine with each other and terminate the active radicals to achieve the purpose of interrupting and inhibiting the combustion of PO, thereby improving flame retardancy [31]. Meanwhile, in the condensed phase, acids, e.g., phosphoric acid (H_3_PO_4_), metaphosphoric acid (HPO_3_), and polymetaphosphoric acid decomposed from inorganic phosphorus FRs, will contribute to the dehydration of PO into carbon. This carbon layer can block the generation of new radicals by limiting the diffusion of oxygen in the combustion environment and protect the underlying polymer from further combustion. At the same time, thermal decomposition reactions will lead to the condensation of phosphoric acid and will release water to decrease the temperature of the matrix and dilute the concentration of combustible materials in the air, thus producing a flame-retardant effect [32].

However, the compatibility of RP with PO matrix is poor, and this adversely affects the mechanical performance of the PO composite. In addition, the low ignition point of RP will be a safety hazard in the application. These factors above lead to the restriction of the RP application.

To solve this issue, RP should be treated before use. An effective method is to encapsulate the RP by using the so-called microencapsulation technology [33,34]. Microcapsule technology is to cover the surface of the FRs with a coating of organic or inorganic materials [35]. After coating, the compatibility of the flame retardant with the substrate can be significantly improved [36]. Liang et al. [37] investigated the influence of the addition of microencapsulated red phosphorus (MRP) on the mechanical performance of PP polymers and found that the elongation at break of PP composites reached a maximum value of more than 900%, with 2 wt.% MRP. However, when the addition of MRP was continued, the elongation at break suddenly decreased, while the tensile strength only slightly decreased (as shown in Figure 6b). This indicates that MRP has a significant effect on the improvement of the mechanical properties of polymer materials at a lower addition.

Moreover, in order to make the polymer achieve higher flame-retardant properties, MRP often needs to be compounded with other FRs, e.g., nanocarbon materials and metal salts [38,39]. Chen et al. [40] used three FRs, i.e., MRP, MH, and Zinc Borate (ZB), in certain ratios for PP polymers. It was shown that 50 wt.% MRP/MH/ZB (6/4/90) could increase the thermal decomposition temperature of PP, thus greatly improving the stability of the composites, while the LOI could reach more than 30%, and the UL-94 test could achieve V-0 grade. Wang et al. [41] microencapsulated RP with an alcohol-soluble phenolic resin to obtain the MRP and used it with aluminum hypophosphite (AHP) for flame-retardant modification of LDPE composites. The results showed that LDPE composites with 10 wt.% MRP could not achieve a V-0 rating; this was because the char layer generated after combustion was insufficiently dense and prone to cracking. Meanwhile, the LDPE/10 wt.% MRP/30 wt.% AHP composite could achieve a V-0 rating and produced a denser carbon layer after combustion. Figure 6c shows the residual carbon shapes of the three LDPE samples. Meanwhile, AHP did not affect the tensile strength of the composite, which still could maintain above 10 MPa with 30 wt.% AHP.

Up until now, microencapsulation has been the most frequently used technique to enhance the compatibility of inorganic phosphorus flame retardants with PO substrates, so as to obtain cable material with excellent flame-retardant and mechanical properties [43,44]. Furthermore, hypophosphite or phosphinate can also serve as useful FRs for the PO matrix due to their characteristics of good thermal stability and high flame-retardant efficiency [45,46]. Tian et al. [47] investigated the effect of AHP on the flame retardancy of LDPE composites. The results showed that the LOI value of the LDPE polymer with 50 phr AHP achieved 27.5%, and it passed the V-0 rating. Moreover, LDPE/AHP composites could still maintain good performance in tensile strength (10.6 MPa). Zhou et al. [48] prepared a batch of EVA/AHP composites with melamine cyanurate (MCA) and MoS_2_ as adjuvants to obtain PO composites with better flame retardancy and mechanical performance. After adding 30 wt.% AHP/MCA/MoS_2_ (18.7/9.4/2), the LOI value of EVA increased to the maximum value of 38.5%, and a V-0 classification was achieved in the UL-94 test. In addition, the elongation at break of the EVA composites increased to 654% after adding 2 wt.% MoS_2_.

In summary, in order to achieve higher flame-retardant efficiency, the development of inorganic phosphorus FRs mainly focuses on the following aspects [49,50,51]: (i) surface treatment—the search for more excellent powder surface modifiers, especially the study of microencapsulation technology; (ii) development of cooperative FRs with better performance—combining different kinds of FRs or introducing different kinds of flame-retardant groups into the same flame-retardant molecular structure to achieve the cooperative effect; and (iii) ultra-fine treatment—develop nanotechnology and carry out research on ultra-fine inorganic phosphorus-based FRs.

### 3.3. Inorganic Silicon

Silicon FRs are divided into organic silicon FRs and inorganic silicon FRs. Inorganic silicone FRs have the characteristics of being non-toxic and having less smoke, low burning value, slow flame propagation, etc. [52]. They include SiO_2_, montmorillonite (MMT), glass fibers, silica gel and talcum powder, etc.

However, the poor compatibility between inorganic silicon FRs and PO substrate will seriously damage the processability and mechanical performance of the composites. Therefore, how to improve the compatibility has become a key issue in the research of inorganic silicon FRs. Micronization (micron, nano, etc.) and surface treatment are common methods so far. Moreover, silicon FRs can be used with other additives to modify PO polymers for flame retardancy and mechanical properties [53]. In general, inorganic silicones are mainly used as adjuvants in combination with other FRs for PO polymers. Among them, SiO_2_ [54,55] and MMT are the most commonly used inorganic silicone adjuvants [56,57]. Thus, this section mainly discusses these two types of FRs.

#### 3.3.1. SiO_2_

SiO_2_ contains many advantages, including small particle size, large specific surface area, and good thermal stability. As an adjuvant for other FRs, it can form a protective layer of silicon on the surface of the material to enhance the flame-retardant effect [58]. The protective layer can play a role in reducing the rate of heat release and improving the mechanical performance of the materials [59].

There are three types of SiO_2_, including fused silica, fumed silica, and silica gel. Kashiwagi et al. [60] explored the different effects of these three types of SiO_2_ as FRs in PP polymers. It was shown that fumed silica and silica gel with a large surface area and a low density can reduce the contact of the polymer with the combustion flame by acting as a thermal barrier layer covering the surface of the molten PP polymer. Thus, they can significantly reduce the total heat release of the PP composite system. However, fused silica, due to its relatively small specific surface area, sinks into the polymer and makes little contribution to thermal insulation and flame retardancy.

Recently, many scholars have conducted research about the SiO_2_ effect on the flame retardancy of PO cable materials [61,62]. Table 2 lists some examples of the combination of SiO_2_ and other conventional FRs. The table compares the effect of SiO_2_ as an adjuvant for flame-retardant system. It is showed that researchers aimed mostly at the EVA polymer. Moreover, with an addition of about 1–5 wt.% SiO_2_, the LOI and tensile strength of the polymers can be improved noticeably.

#### 3.3.2. MMT/OMMT

**Figure 7:**
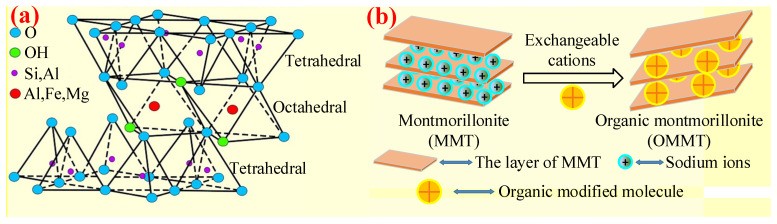
(**a**) The structure of MMT. Reprinted from Reference [68] with permission. (**b**) Schematic diagram of montmorillonite organic modification.

MMT is a very soft nano-layered silicate consisting of a central sheet of alumina octahedral and two sheets of silica tetrahedral. Accordingly, its structure is shown in Figure 7a, displaying two layers of stacked sheets [68]. MMT is favored for its abundant mineral resources, excellent flame-retardant properties, and low price, so it has become an important inorganic additive in the field of flame retardants. Studies showed that a small amount of MMT had a positive effect on improving the thermal stability, mechanical performance, and flame-retardant properties of PO substrates [69,70]. Meanwhile, MMT has the advantages of low smoke and non-toxicity. As an adjuvant, it has great application prospects for flame-retardant PO cable materials [71,72].

However, since the PO matrix is a typical nonpolar polymer material, while the surface of MMT sheet layer is highly hydrophilic and polarized, the compatibility between them is poor and cannot achieve good dispersion, thus seriously affecting the mechanical performance of composites. In order to solve the above problems, one approach is to functionalize PO by introducing polar or polarizable groups into the polymer to convert the nonpolar molecular chains of PO into polar ones [73]. Another commonly used method, as the focus of this section, is to obtain organic montmorillonite (OMMT) by making organic matter enter MMT sheets via exchanging with interlayer inorganic cations, hydrophobizing the hydrophilic MMT surface, and reducing the surface energy of MMT, as shown in Figure 7b, which has good compatibility with PO [74,75].

Table 3 shows the effect of MMT/OMMT as adjuvants for a flame-retardant system. It is shown that, for most PO/FRs composites, after adding a small amount of MMT/OMMT (1–5 wt.%), the flame retardancy and mechanical properties are improved. It is observed that the mechanical properties of the materials can be greatly improved by replacing MH/ATH with a small amount of OMMT/MMT; this may be due to the fact that the latter is easier to disperse in the PO matrix.

## 4. Organic Flame Retardants

### 4.1. Organic Phosphorus

According to the element types and molecular structures, organic phosphorus FRs are divided into three types: phosphorus-containing esters (including phosphate esters, phosphite esters, metaphosphate esters, phosphonates, hypophosphonates, etc.) [86,87], phosphorus–nitrogen adjuvants (e.g., phosphonitrile) [88,89,90], and phosphorus–silicon adjuvants [91,92]. The flame-retardant mode of action of organic phosphorus in PO polymer is the same as that of inorganic phosphorus FRs, including both a gas-phase and condensed-phase effect [93,94].

**Figure 8:**
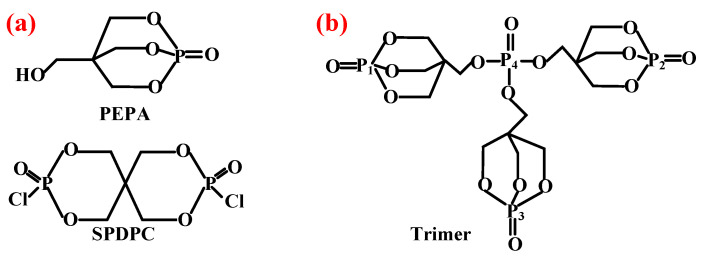
(**a**) Structures of PEPA and SPDPC. Reprinted from References [95,96] with permission. (**b**) Chemical structure of Trimer.

As for the phosphorus-containing esters FRs, caged bicyclic phosphate PEPA (1-oxo-4-hydroxymethyl-2,6,7-trioxa-l-phosphabicyclo [2.2.2] octane) [95] and SPDPC (spirocyclic pentaerythritol bisphosphorate disphosphoryl chloride) [96] are important intermediates for the synthesis of them (shown in Figure 8a). Moreover, a series of FRs with high phosphorus content can be developed by grafting other functional groups with flame-retardant properties on the double ring cage structure of PEPA and the spiral ring structure of SPDPC. Li et al. [97] used PEPA and phosphorus oxychloride to synthesize a novel phosphate flame retardant with a structure of caged bicyclic, tri(1-oxo-2,6,7-trioxa-1-phosphabicyclo12.2.2] octane-methyl) phosphate (Trimer) (in Figure 8b). It was shown that the Trimer molecule contained three bicyclic caged phosphate units with a high phosphorus content of 21.2 wt.% and had a highly symmetric structure, resulting in high char-forming properties and thermal stability. Jiang et al. [98] mixed Trimer and APP in the weight ratio of 2:1 to obtain a mixture as the flame retardant for PP matrix. When 25 wt.% of them was added, the LOI value reached 28.8% with V-0 grade in UL-94 test. Moreover, Wang et al. [99] synthesized a novel phosphorus-containing flame retardant (SPDH) via the interaction of synthesized intermediate product SPDPC with 10-(2,5-dihydroxyphenyl)-9,10-dihydro-9-oxa-10-phosphaphenanthrene-10-oxide (DOPO-HQ). When the content of SPDH was 40 wt.%, an LOI value of 24.6% and a V-0 classification in UL-94 test were achieved.

**Figure 9:**
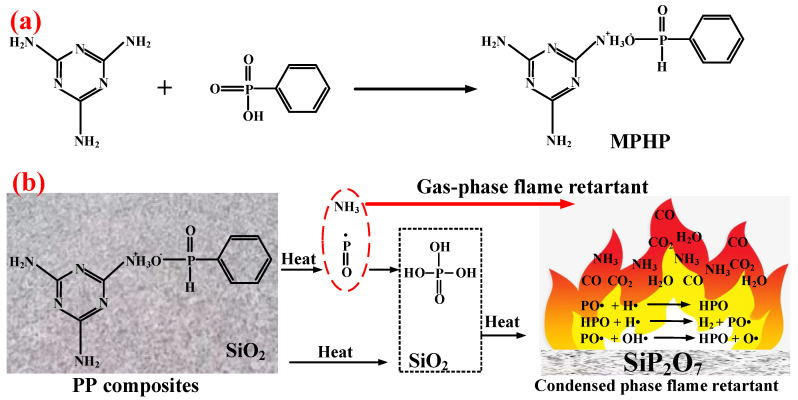
(**a**) Synthesis route of MPHP. (**b**) Model of phosphorus–silica cooperative flame-retardant PP composites. Reprinted from Reference [62] with permission.

Polyphosphazene, as a kind of phosphorus–nitrogen adjuvant, has excellent flame retardancy and thermal stability. Due to its special molecular structure and flexible main chain, polyphosphazene can play a positive role in improving the mechanical properties of polymers [100]. Wu et al. [101] synthesized a new phosphazene derivative, i.e., hexakis(dodecylamino)cyclotriphosphazene (H-12), as an adjuvant for flame-retardant PP/Trimer/APP composites. The results showed that H-12 could enhance the compatibility of Trimer/APP with EVA polymer. Thus, it improved its flame-retardant properties and mechanical performance. By replacing 10 wt.% content of Trimer/APP with the same amount of H-12, the elongation at break of the sample could reach more than 900%, with an LOI value of 29.1%. Furthermore, Ai et al. [62] synthesized an organic compound containing phosphorus and nitrogen, i.e., MPHP, which also belongs to the phosphorus–nitrogen adjuvants, with its synthesis method shown in Figure 9a. They studied the influence of MPHP on the mechanical performance and flame-retardant properties of the PP matrix. The obtained results showed that, when the PP/FRs composite contained 30 wt.% MPHP, the LOI value went up to 31.3%, and the UL-94 test achieved V-0 grade. Moreover, the tensile strength of the material was 23.4 MPa. Afterward, 3 wt.% SiO_2_ was added in the PP/30% MPHP composites as an adjuvant. Figure 9b shows that a large number of phosphorus-containing radicals and ammonia gas will generate from MPHP after thermal decomposition. Among them, ammonia gas can reduce the concentration of combustible gas in the air, and this has a positive effect on inhibiting the burning intensity of PP matrix, while phosphorus-containing radicals interrupt the chain reaction by consuming radicals, e.g., H·and ·OH, thereby inhibiting the decomposition of PP. Meanwhile, in the condensed phase, a heat-resistant carbon layer containing SiP_2_O_7_ was formed to cover on the surface, so that MPHP/SiO_2_ FRs improved the LOI values of PP composites.

### 4.2. Organic Silicon

Organic silicon has always been in the frontline of co-additives in FRs systems for PO composites because of its characteristics of high efficiency, low smoke evolution, and environmentally friendliness [102]. Organic silicon includes silicone oil, silicone rubber, polysiloxane, and its derivatives. Their flame-retardant mode of action is that, when the PO composite is burning, the organic silicone can migrate to the outer surface of materials. The -Si-O- bonds in its molecule will be transformed into -Si-C- bonds. Then the generated white combustion residue and carbide form a composite inorganic layer covering the material to prevent the combustion volatiles from escaping and block the oxygen from contacting the substrate [103]. In addition, organic silicone FRs can significantly improve the mechanical performance of PO cable materials, because they can enhance the interface force between the FRs and the matrix [104].

There are two types of organic silicones used as FRs for PO substrates, i.e., additive type and reactive type. The former means that organic silicone is added to the PO substrate by itself or with other FRs without forming chemical bonds with PO matrix. The reactive type mainly refers to the preparation of new flame-retardant polymers by copolymerizing with flame-retardant units and grafting or crosslinking with macromolecules [105,106,107]. However, compared with reactive type of organic silicones, much more research has been conducted on the additive type to improve the flame-retardant properties. Therefore, the influence of additive-type silicone FRs on PO polymer is the focus of this section.

Thus far, researchers have tried a series of halogen-free flame-retardant additives to improve the mechanical performance of PO substrates. Nevertheless, the experimental results rarely showed a good balance between mechanical performance and flame retardancy [108,109]. To overcome this problem, FRs can be modified by surface treatment techniques with organic silicon, which has a significant effect on improving the interfacial adhesion between the flame-retardant additive and the polymer matrix [110,111]. Previously, the traditional reagents used for surface treatment were mainly silane coupling agents, but their effects were limited [112,113]. Recently, polysiloxane and its derivatives have become significant surface modifiers of FRs used for PO polymers because of their high flame-retardant efficiency, high thermal stability, and excellent compatibility with PO substrates [114,115].

Table 4 presents the results regarding the influence of additive type of organic silicon FRs on the performance of the PO matrix. It can be seen that most research focuses on PP polymers, and silicones are mostly used to modify polar APP, thereby improving the compatibility of APP with the polymer matrix. It is found that both the flame retardancy and mechanical properties of the material are improved by adding silicone as an adjuvant.

## 5. Intumescent Flame Retardants

The mode of action of IFR is to add some intumescent additives as flame retardants in the preparation of PO materials that will decompose into an intumescent carbon layer wrapping around the surface of the PO matrix under high-temperature conditions, playing a role in isolating heat, combustible volatile gases, and oxygen, thus blocking the combustion process [124,125].

In the development process of developing polymers that are flame retardant, phosphorus/nitrogen-based intumescent flame retardants (P-N based IFR), first pioneered by Camino et al. [126] in 1990, have played a great role. These FRs have the characteristics of high flame-retardant efficiency, low smoke, and low negative impact on the mechanical performance of composites. A conventional IFR system consists of three components: APP, MEL, and PER [127,128]. Later, in 1994, Fukuda et al. [129] first used expandable graphite (EG) for flame-retardant PO materials. They found that EG is highly resistant to corrosion. Moreover, EG has good durability, and it is environmentally friendly [130]. Since then, EG has become an important member of the family of expandable flame-retardant materials.

### 5.1. P-N-Based IFR

**Figure 10:**
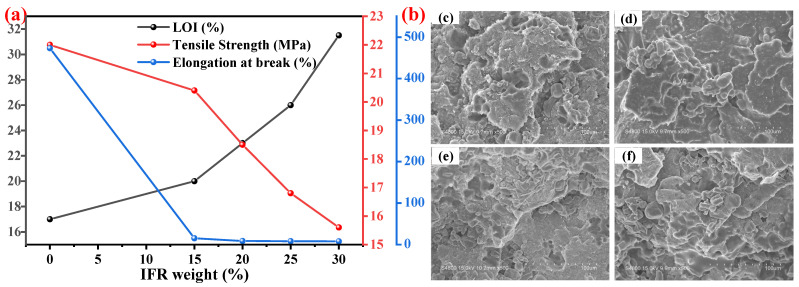
(**a**) Comparison of elongation at break/tensile strength and LOI. (**b**) SEM images of HDPE/IFR composites: (**c**) HDPE/15 wt.% IFR, (**d**) HDPE/20 wt.% IFR, (**e**) HDPE/25 wt.% IFR, and (**f**) HDPE/30 wt.% IFR. Reprinted from Reference [132] with permission.

P-N-based IFR is the most abundant and most widely used FR in the IFR systems. The MEL, APP, and PER components are used as the gas source, acid source, and carbon source, respectively [131]. However, conventional P-N-based IFRs cannot be uniformly dispersed in the PO matrix due to the large addition amount and polarity difference. As a result, the mechanical properties of the material are seriously affected. Khanal et al. [132] used an IFR system consisting of APP and tris (2-hydroxyethyl) isocyanurate (THEIC) to prepare flame-retardant HDPE composites, whose mechanical performance and flame retardancy were investigated. The trends of flame retardancy and mechanical performance of the composites, e.g., LOI, tensile strength, and elongation at break values, With the increase of IFR contents are shown in Figure 10a. It was observed that, within a certain range, with the increase of the flame-retardant addition, the LOI value increased, while the elongation at break and tensile strength showed a significant downward trend. They proposed that, since the IFR contains polar components, while the polymer matrix is a nonpolar material, the difference in polarity between them leads to poor compatibility, which can be explained by the obvious gaps existing between the IFR and the matrix, as shown in Figure 10b.

In response to the above problems, researchers have conducted a large number of experimental studies, including the surface treatment of P-N-based IFRs, the addition of cooperative FRs, and the synthesis of new IFRs. In the next section, the research progress in these three directions is described in detail.

#### 5.1.1. Surface Treatment of IFR

•Microencapsulation

The different polarities between the IFR and PO matrix leads to the poor compatibility of IFR, which severely reduces the mechanical properties of the PO composites [133]. Up to now, microencapsulation technology has been considered one of the effective strategies to enhance the compatibility of IFR. The microencapsulated IFR with a core–shell structure is isolated from the surrounding material, and this can improve its compatibility with polymer matrix [134,135]. In general, there are several kinds of materials that can be used as encapsulating shells for IFR and that are prepared by the methods of physics encapsulation or in situ polymerization, e.g., MF, silicon resin, melamine, urea-melamine-formaldehyde, or polyurethane (PU) [136,137,138,139].

**Figure 11:**
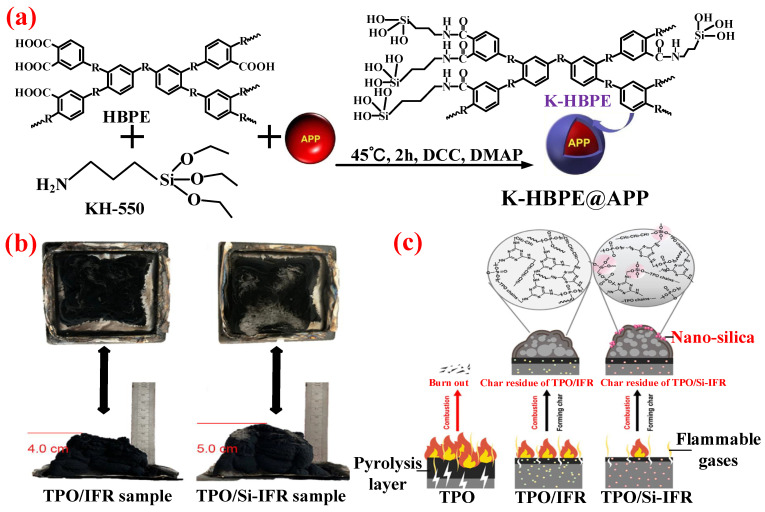
(**a**) The synthetic route of K-HBPE@APP. Reprinted from Reference [140] with permission. (**b**) The photos of the char residues of the TPO sample. Reprinted from Reference [141] with permission. (**c**) Flame-retardant mode of action. Reprinted from Reference [141] with permission.

Zhang et al. [140] used HBPE, a hyperbranched polyester, to microencapsulate APP via KH-550 (shown in Figure 11a) and investigated the flame-retardant effect of the organic–inorganic hybrid K-HBPE@APP in PP. It was shown that, compared to adding equal amounts of K-HBPE and APP, the addition of K-HBPE@APP not only increased the UL-94 rating (from V-1 to V-0) and the LOI value (from 31.0% to 34.2%), but also exhibited an obvious effect on improving the elongation at break (from 83% to 375%) and tensile strength (more than 20 MPa) of the IFR/PP materials. The abovementioned improvement is mainly attributed to the more uniform dispersion of the microencapsulated HBPE and APP in the PP matrix. Wang et al. [141] adopted a synthesized silicone resin called poly-DDPM to encapsulate IFR additives and then incorporated the obtained Si-IFR into thermoplastic polyolefins (TPO) to improve the flame retardancy and mechanical performance. The results showed that the TPO/20 wt.% Si-IFR achieved the V-0 rating in the UL-94 test, along with an LOI value of 32.2%. However, when adding the same amount of untreated IFR, the LOI value was only 29.6%. Meanwhile, the elongation at break and tensile strength reached 780% and 11.3 MPa from 750% and 10.3 MPa, respectively. Therefore, TPO/Si-IFR exhibited excellent balances between flame retardancy and mechanical performance. As shown in Figure 11b, TPO/Si-IFR produced a larger volume of expanded carbon layer after combustion compared with TPO/IFR. The possible flame-retardant mode of action of Si-IFR is proposed as follows: (i) APP/charring–foaming agent (CFA) promoted the crosslinking and rearrangement of pyrolyzed TPO chains, which protected the TPO from further burning; and (ii) degradation of poly-DDPM generated nano-silica, which migrated to the surface during TPO combustion and enhanced the integrity and quality of the carbon layer (shown in Figure 11c).

•Surface modification

Surface modification is another feasible method to get rid of the drawback of incompatibility between IFR and PO. A number of modifiers were applied to promote the interfacial compatibility, such as KH-550, KH-560, and silicone oil [142,143], which combined the functions of coupling effects and dispersing effects to bond the IFRs and PO matrix by chemical bonds. As an indispensable component of traditional P-N-based IFRs, APP can act as both an acid source and gas source. However, APP itself belongs to inorganic material, and PO polymer is organic material, and there is inevitably a problem of poor compatibility between them in direct blending, which leads to a great reduction of mechanical properties of composite materials [144].

**Figure 12:**
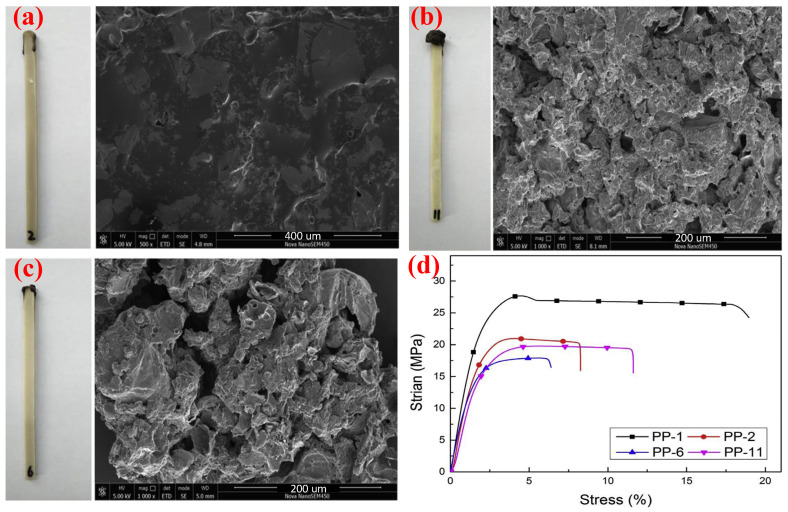
The actual combustion result photographs and SEM of residual char: (**a**) PP/30 wt.% APP, (**b**) PP/20 wt.% APP/10 wt.% UF, and (**c**) PP/20 wt.% APP/10 wt.% M-UF. (**d**) The strain–stress trend of PP composites (PP-1, pure PP; PP-2, PP/30 wt.% APP; PP-6, PP/20 wt.% APP/10 wt.% UF; and PP-11, PP/20 wt.% APP/10 wt.% M-UF). Reprinted from Reference [146] with permission.

To solve this problem, Lin et al. [145] used the APP, which was modified by KH-550 as FRs for the PP matrix. The results showed that this method improved the LOI values (from 16.0% to 30.0%). Furthermore, the obtained composite had excellent mechanical properties; when 20 wt.% modified APP was added, the elongation at break was 308%, and the tensile strength was 26.9 MPa. Meanwhile, the values of them with unsurfaced IFR were only 269% and 21.6 MPa. Moreover, Ren et al. [146] firstly synthesized urea-formaldehyde resin (UF) and then modified it with KH-550 to obtain M-UF. They studied the carbon-formation effect of M-UF and used M-UF in combination with APP as a flame retardant for PP polymers. As illustrated in Figure 12a–c, compared with the two composites PP/30 wt.% APP and PP/20 wt.% APP/10 wt.% UF, the sample of PP/20 wt.% APP/10 wt.% M-UF had more intumescent carbon layer, of which the surface was more compact and consecutive with less holes, so it had a better flame-retardant performance reflected in both UL-94 testing grades and LOI values. The surface modification of IFR increased the elongation at break of the composite by 104% compared to the previous one (as shown in Figure 12d).

In addition, the methods of modification for IFRs have been mentioned in other studies, as given in Table 5. It can be seen that PP polymers are the research focus. Compared with the untreated IFR system, the surface-treated IFR is beneficial to further improve the LOI value and elongation at break for the PO polymers.

#### 5.1.2. Adjuvants for IFR

In addition, researchers found that by adding other FRs, e.g., metal hydroxides, carbon nanomaterials, or silicon-based materials, to replace part of the IFR, the mechanical properties of the PO materials could be improved, while ensuring the flame retardancy. Firstly, these adjuvants make the release of non-combustible gases more stable during the IFR combustion process, thus helping PO polymers to form a dense and stable expanded carbon layer. Secondly, they can also be used as supplements for IFR to enhance the gas phase and condensed phase barrier effects, thereby reducing the risk of combustion in composites and positively impacting the mechanical performance of PO polymers.

•Metal-based adjuvants

Metal-based compounds have the function of accelerating the dehydration of PO matrix and IFR to form a compact and stable carbon layer. Moreover, a small amount of metal-based adjuvants added into the PO/IFR cable materials can have a positive effect on the flame-retardancy efficiency.

**Figure 13:**
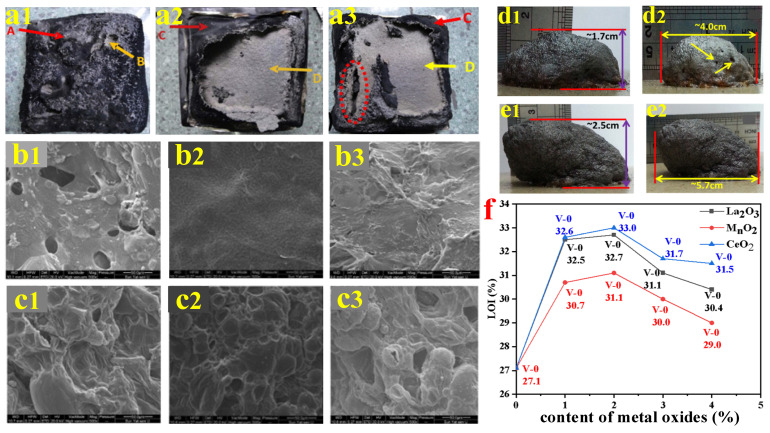
(**a1**–**a3**) Digital pictures for chars of PP/IFR (**a1**), PP/IFR/1 wt.% La_2_O_3_ (**a2**), and PP/IFR/1 wt.% MnO_2_ (**a3**), A and B are outer and inner surface of char residue of PP/IFR, and C and D are outer and inner surface of char residue of PP/IFR/1 wt.% La_2_O_3_ and PP/IFR/1 wt.% MnO_2_. (**b1**–**c3**) SEM images of char residue for PP/IFR (**b1**,**c1**), PP/IFR/1 wt.% La_2_O_3_ (**b2**,**c2**), and PP/IFR/1 wt.% MnO_2_ (**b3**,**c3**) ((**b1**–**b3**): outer 500×, (**c1**–**c3**): inner 500×). (**d1**,**d2**) Digital pictures for IFR (**e1**,**e2**) and IFR/CeO_2_ (**e1**,**e2**) heated at 500 °C for 5 min. (**f**) Influence of metal oxides content on the performance of flame retardancy for PP/IFR composites. Reprinted from References [155,156,157] with permission.

Feng et al. [155,156,157] studied the synergism of three metal oxides (La_2_O_3_, MnO_2_, and CeO_2_) on the carbon-forming mode of action of PP/IFR composites. It was found that these three metal oxides promoted PP/IFR systems to form a more continuous and intensive intumescent carbon layer (shown in Figure 13(a1–e2)) and significantly enhanced the LOI value of the PP/IFR composites. Meanwhile, the materials passed a V-0 grade in the UL-94 test. As shown in Figure 13f, with the increase of the content of these three metal oxides, the LOI value and UL-94 grade of the PO polymer showed a trend of first increase and then decrease. When the addition was 2 wt.%, the compounds achieved the best flame-retardant properties. Furthermore, Qin et al. [158] investigated the influence of nano-ATH on the flame retardancy and mechanical performance of the PP/IFR. It was shown that 2 wt.% nano-ATH helped the LOI value of the PP/IFR composites increase from 26.6% to 31.2%, with a V-0 grade in the UL-94 test. Nano-ATH was found to be a very effective cooperative agent in the PP/IFR system, as it could catalyze the chemical reactions of carbon components and acid components to form a dense, hard char layer covering the surface of the polymer. Moreover, 2 wt.% nano-ATH increased the elongation at break and tensile strength of PP/IFR composites from 15% and 24.2 MPa to 41% and 25.5 MPa, respectively.

Moreover, it was found that transition metal ions, such as Fe^3+^, Zr^4+^, Sr^2+^, Zn^2+^, etc., can also be used as catalysts for reactions of dehydrogenation and crosslinking of polyolefin substrates [159]. Chen et al. [160] studied the cooperative influence of strontium carbonate (SrCO_3_) as the cooperative agent on the mechanical performance and flame retardancy of PP/IFR system. The cooperative effect could be observed by the enhanced LOI value (from 36% to 36.1%) and UL-94 grade (from V-1 to V-0) of PP/IFR composites with 1.5 wt.% SrCO_3_. Moreover, the tensile strength of PP composites enhanced from 26.8 to 29.4 MPa compared with the sample without the SrCO_3_ addition. It was suggested that, on the one hand, SrCO_3_ could catalyze the chemical reaction between MAPP and PER, thereby promoting the PP polymer to form a stable and dense carbon layer structure. On the other hand, for the phosphate existing in the carbon layer, SrCO_3_ could generate bridge bonds between them, forming a crosslinked char layer rich in P element, which further improves the stability of the carbon layer. Therefore, the appropriate amount of SrCO_3_ also contributes to the improvement of mechanical performance of PP composites.

•Carbon-based adjuvants

The preparation of polymer/nano-carbon composites has become an effective method to enhance the flame retardancy of materials [161,162]. There have been numerous studies in the literature published which focus on the effect of carbon nanomaterials on the performance of polymers, for instance, carbon nanotubes (CNTs) [163,164], graphene [165,166], and carbon black (CB) [167,168]. Carbon-based adjuvants have become one of the most promising “green” flame-retardant additives, with the advantages of low smoke, no halogen, and high efficiency. Their flame-retardant mode of action is mainly attributed to its large specific surface area and the ability to create a better carbon layer in condensed phase through chemical reaction with APP during combustion, which increases the “barrier effect” and effectively blocks the heat transfer and diffusion of combustible materials, playing a protective role for the PO matrix [169,170]. In addition, a small amount of carbon nanomaterials can significantly decrease the combustibility of polymers and improve the mechanical properties due to the high mechanical strength and stiffness of these carbon-based adjuvants [171].

**Figure 14:**
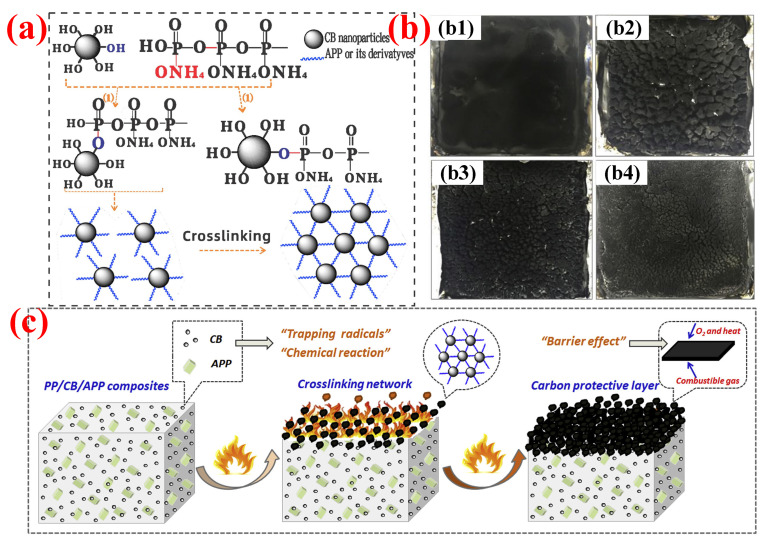
(**a**) Possible chemical reaction between CB and APP to form crosslinking network during combustion. (**b**) Morphology of residual chars from PP composites after cone calorimeter tests from (**b1**) 25APP, (**b2**) 3CB/22APP, (**b3**) 5CB/20APP, and (**b4**) 7CB/18APP. (**c**) Schematic representations of flame retardancy mode of action of CB and APP in PP system. Reprinted from Reference [172] with permission.

Yang et al. [172] investigated the cooperative effect of nano-CB and APP in PP. Their cooperative interaction could be observed by the increased LOI value (29.8%) and UL-94 grade (V-0), under the optimum specific gravity corresponding to 18 wt.% APP and 7 wt.% nano CB. However, when adding 25 wt.% APP, the corresponding LOI and UL-94 grade of the polymer were 20.9% and no rating. Figure 14a shows the chemical reaction between nano-CB and APP during combustion and the formation of a crosslinked network, which helps to strengthen the structure of the carbon protective layer. As a result, it is possible to obtain expanded char layers with different carbon contents and compactness by adjusting the addition of APP and nano-CB (shown in Figure 14b). The polymers with thick and dense carbon residue correspond to a high flame-retardancy efficiency. Figure 14c shows the schematic diagram of the flame-retardant mode of action of APP and nano-CB in PP system. On the one hand, a chemical reaction occurred between APP and nano-CB to form a crosslinked network structure, which can promote the formation of a more stable carbon layer in the condensed phase. On the other hand, the “trapping radicals” of nano-CB and APP derivatives could delay or even restrain the degradation of PP. Wen et al. [173] studied the influence of nanosized CB as adjuvant on the flame retardancy and mechanical performance of PP/POE-MA (maleic anhydride-grafted polyolefin elastomer)/IFR system. They found that, within a certain range, as the nano-CB content increased, the char layer produced by polymer combustion became increasingly dense and continuous. Moreover, the PP composites reached the LOI value of 29.7% from 26%, with the V-1 grade in the UL-94 test, under the addition of 3 wt.% nano-CB and 8 wt.% POE-MA. Meanwhile, POE-MA and nano-CB, as a toughening agent and rigid nanoparticles, respectively, showed significant improvement in elongation at break and tensile strength, reaching 215% and 32.8 MPa, respectively.

•Silica-based adjuvants

Recently, silica-based FRs have attracted extensive attention in the research on cooperative flame-retardant PO/IFR composites, such as SiO_2_ [174,175], MMT (OMMT) [176,177], and polysiloxane [147,178] https://www.x-mol.com/paperRedirect/1296136865981276160.

**Figure 15:**
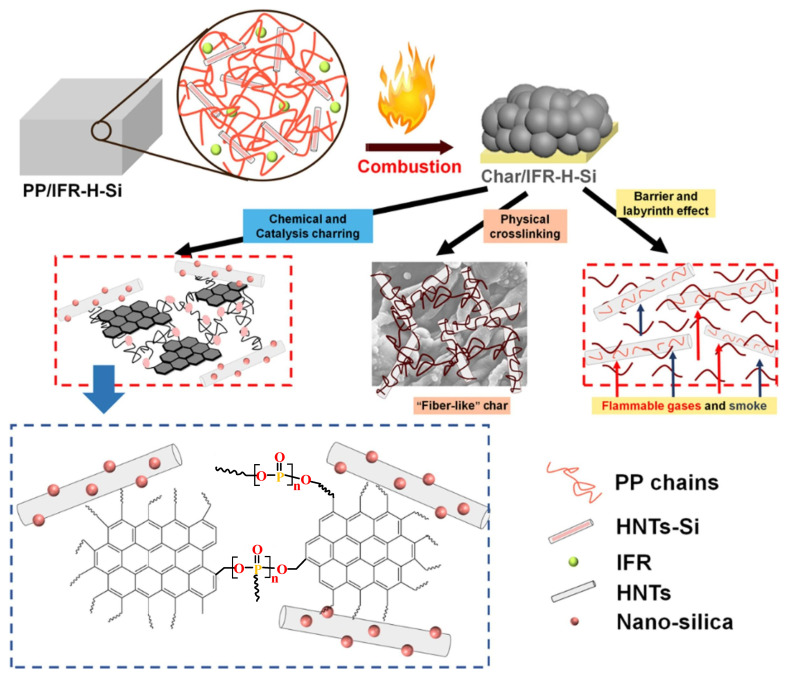
The carbonization mode of action of HNTs-Si in PP/IFR composites. Reprinted from Reference [181] with permission.

Wen et al. [179] used OMMT as an adjuvant for the IFR constructed with a hyperbranched charring foaming agent (HCFA) and APP to achieve better flame retardancy for the PP matrix. It was found that a proper amount of OMMT (2 wt.%) dramatically enhanced the LOI value of PP/20 wt.% IFR from 29% to 31.5% and made it V-0 grade in the UL-94 test. Meanwhile, Yang et al. [180] confirmed the cooperative influence of octahedral polyhedral sesquisiloxane (OV-POSS) on PP/IFR composites. The incorporation of OV-POSS obviously enhanced the dispersion of IFR in the PP substrate and the compatibility between them, thus improving the flame retardancy and mechanical performance of PP/IFR composites. Wang et al. [181] filled kaolinite nanotubes with polysiloxane to obtain HNTs-Si and introduced 1.2 wt.% HNTs-Si into PP/IFR to obtain polymer materials with excellent flame retardancy and mechanical properties. The presence of HNTs-Si made the UL-94 test achieve the V-0 level without ignition, and the LOI was further increased to 30.6% from 29.1%. As shown in Figure 15, the cooperative effect of HNTs-Si and IFR promotes the formation of dense and crosslinking carbon layers, which can cover the PP matrix to effectively prevent the release of flammable gases and enhance the flame retardancy of PP composites. Meanwhile, the addition of HNTs-Si improves the extensibility of PP/IFR composites, of which the elongation at break increased from 8.6% to 45.4% because of the bridging effect between the PP matrix and HNTs.

•Other adjuvants

**Figure 16:**
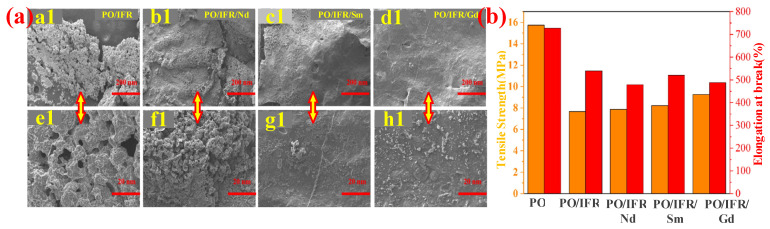
(**a**) SEM images ((**a1**–**d1**) low magnification and (**e1**–**h1**) high magnification) of PO materials. (**b**) Trend of elongation at break and tensile strength with RES content. Reprinted from Reference [182] with permission.

The three types of adjuvants above added to the PO cable materials have achieved significant cooperative improvements in flame retardancy and mechanical properties. For instance, Jia et al. [182] prepared a series of rare-earth stannates, i.e., Re_2_Sn_2_O_7_ (RES, Re = Nd, Sm, and Gd) via the hydrothermal method, which can be applied as an adjuvant for PO/IFR composites due to its high-temperature catalytic performance. Moreover, they thoroughly studied the flame retardancy and mechanical performance of the PO/IFR/RES composites. The results showed that RES could enhance the UL-94 classification and LOI value of PO/IFR composites. The LOI of PO/IFR composites increased from 30% to 34%, 33%, and 32%, after adding Nd_2_Sn_2_O_7_, Sm_2_Sn_2_O_7_, and Gd_2_Sn_2_O_7_, respectively. The mode of action of flame retardancy is that the PO/IFR/RES composites can form a more continuous and compact protective carbon layer (shown in Figure 16a), which can protect PO substrates from the influence of oxygen and heat and effectively suppress further degradation of PO substrates [183,184]. At the same time, RES allows PO/IFR composites to maintain good mechanical properties (in Figure 16b). These three adjuvants (Nd_2_Sn_2_O_7_, Sm_2_Sn_2_O_7_ and, Gd_2_Sn_2_O_7_) all improved the tensile strength of PO/IFR, but they have no obvious effect on the elongation at break.

Moreover, there are various additives used as adjuvants with different IFRs, which are summarized in Table 6. It can be seen that most research focuses on PP polymers. Moreover, most of adjuvants were devoted to improving the flame-retardant performance, and the effect is very significant.

#### 5.1.3. New IFRs

In addition to the methods of surface treatment and cooperative additives, there is another feasible solution to enhance the distribution of IFR in the PO matrix, i.e., designing a single-component IFR by combining the components of acid, carbon, and gas into a macromolecular structure, the so-called trinity IFR [198,199,200].

**Figure 17:**
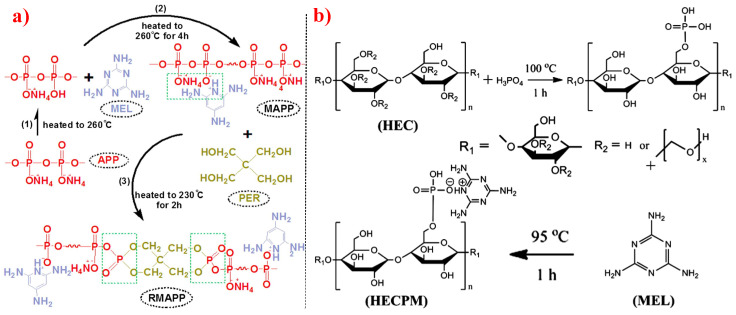
(**a**) Synthesis schematic of RMAPP. Reprinted from Reference [180] with permission. (**b**) Synthesis schematic of HECPM. Reprinted from Reference [201] with permission.

Based on the conventional IFR system of APP/MEL/PER, Yang et al. [180] synthesized a novel trinity intumescent flame-retardant RMAPP (the schematic diagram is shown in Figure 17a) and introduced it to PP with OV-POSS. The PP/RMAPP/OV-POSS achieved an LOI of 31.3%, V-0 classification in the UL-94 test, and tensile strength of 30 MPa. Meanwhile, Zheng et al. [201] prepared a mono-component IFR named HECPM, which had a cellulose-based structure grafted with phosphate groups and melamine groups (shown in Figure 17b) and applied it to the PP/IFR system, with EG as an adjuvant. The obtained results showed that the PP composites reached 31.5% of LOI value and passed the V-0 grade in the UL-94 test when they were mixed with 30 wt.% HECPM and 22.5 wt.% EG. Moreover, Huang et al. [202] synthesized a biobased IFR (PIMEPA@ATH) via metal chelation and used it for the EVA matrix. It was proposed that EVA with 35 wt.% PIMEPA@ATH possessed good flame retardant efficiency and kept good mechanical properties with the elongation at break over 850%. Qi et al. [203] used a novel single-component IFR named PSTBP, i.e., poly(spirocyclic pentaerythritol bisphosphonate-1,3,5-triazine-O-bicyclic pentaerythritol phosphate), to improve the flame retardancy of PP. The results demonstrated that PP/30 wt.% PSTBP mixture could attain an LOI value of 32.5% with a V-0 rating. Xia et al. [204] prepared a trinity IFR (PPMPNG) via neopentyl glycol, piperazine, and methylphosphonic acid to improve the performance of PE matrix. Results showed that PPMPNG showed flame retardancy by quenching reactive radicals in gas phase and by exerting isolation function in condensed phased. Gao et al. [205] used MEL, polyphosphoric acid and THEIC as raw materials to prepare an integrated IFR (TPM). When 25 wt.% IFRs were added into the PP polymer, it achieved V-0 classification in the UL-94 test, and the LOI value increased to 29.3%. Furthermore, the PP/TPM composite had a better mechanical performance than PP/APP/PER. In summary, the single-component IFR should be one of the trends in the field of IFRs [206].

Furthermore, designing new molecules as acid sources or carbon sources for IFR systems also has a certain contribution to improve the performance in flame retardancy and mechanical properties of materials. Table 7 summarizes papers about the new molecules or their compounds as acid sources or carbon agents in recent years.

### 5.2. Expandable Graphite

EG is a layered crystalline carbon-atom-embedded compound. The acid ions between the layers will be released when heated, causing EG to dehydrate and carbonize [130], thus forming a compact and worm-like carbon layer covering the surface [225]. However, the charred layer formed during combustion is very loose and easily falls off due to the “popcorn effect”. Thus, a number of investigations are focused on cooperative effects of EG with other additives to overcome this disadvantage [226], such as APP [227,228,229], MH [230,231], and LDH [232,233].

**Figure 18:**
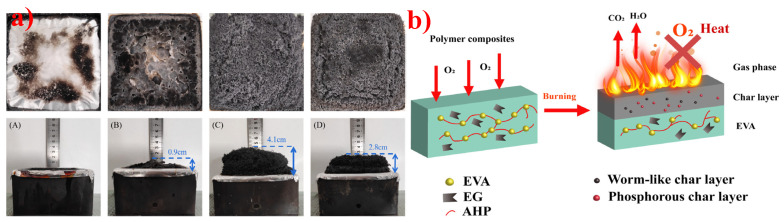
(**a**) The digital photos of carbon residues: EVA (**A**), EVA/15AHP (**B**), EVA/15EG (**C**), and EVA/10EG/5AHP (**D**). (**b**) Schematic diagram of flame-retardant mode of action for EVA/EG/AHP. Reprinted from Reference [234] with permission.

When Yang et al. [234] used AHP as the adjuvant for EG/EVA composites, the EVA/10 wt.% EG/5 wt.% AHP achieved a V-0 grade in UL-94 test, and the LOI value reached 30.5% from 26.5% after the addition of 5 wt.% AHP. Meanwhile, the elongation at break and tensile strength of EVA composites reached 732% and 12.2 MPa, respectively. Figure 18a indicates that the cooperative effect of EG and AHP can promote the carbonization of the EVA polymer and thus provide a good flame-retardant performance. Moreover, Figure 18b shows the mode of action of AHP and EG for flame-retardant EVA; they can exert flame-retardant effects in both gas and condensed phases.

Furthermore, various studies on the cooperative flame-retardant effect between EG and other additives are listed in Table 8. It can be concluded that the cooperative effect of EG and other FRs can greatly improve the flame-retardant and mechanical properties of the materials. Moreover, EG can increase the LOI value of PO polymers to more than 30% with about 40 wt.% metal hydroxide, but it will greatly reduce the elongation at break. For this purpose, elastomeric polymers, e.g., POE and EVA, can be added to improve the flexibility of the material.

## 6. Summary and Perspectives

In this article, the FR compounds for PO cable sheath materials were reviewed, with emphasis on the cooperative modification of flame retardancy and mechanical properties. For the three typical PO materials, i.e., PP, EVA, and LDPE, Table 9 summarizes the FRs used for modification in the literature with the best cooperative performance of flame retardancy and mechanical property. It can be seen that LDPE shows overall properties not as good as PP and EVA, thus indicating that it is more challenging to develop FRs for LDPE while sustaining mechanical performance.

Due to environmental consideration, more research will be focused on the halogen-free flame-retardant PO cable sheath materials in future. The FR compounds can be divided into inorganic FRs, organic FRs, and IFRs, while the IFRs include both organic and inorganic compounds. Regarding the flame-inhibition strategies, they can be divided into two mechanisms: gas-phase FR mechanism and condensed-phase FR mechanism. However, in many situations, the FRs affect the mechanical performance of the PO matrix due to the poor compatibility between them, especially when a higher loading of FR compounds is utilized. Therefore, when designing a FR formulation for PO cable sheath materials, the comprehensive performance of both flame retardancy and mechanical performance should be considered. To address the compatibility of the PO matrix/flame retardant compounds and enhance the adhesion between them, a series of methods have been adopted, including surface treatment, i.e., microencapsulation and surface modification; ultra-fine treatment; and cooperative combinations of different FRs. Among them, surface treatment techniques are often used in inorganic FR composites, e.g., metal hydroxide, inorganic phosphorus, and inorganic silicon, to overcome the polarity difference between them and the PO matrix, thereby improving the adhesion properties between the two interfaces. At present, the nanoscale FRs obtained by ultra-fine processing are mainly silicon-based materials (e.g., MMT and OMMT), carbon-based materials (e.g., C_60_, CNTs, and graphene), and metal hydroxide (e.g., nano-ATH and nano-MH). By adding these nanoscale FRs, PO cable sheath materials with improved mechanical properties can be obtained. Furthermore, the use of different kinds of nanoscale FRs or their combination with other P-, N-, or Si-containing FRs is a promising strategy to develop flame-retardant polymer composites with enhanced mechanical properties. In addition, among the FRs used in PO, IFR is attracting more attention due to its good efficiency and environment friendliness. However, there are disadvantages in the traditional IFR system, such as the incompatibility with PO matrix and the high loading, which would make it difficult for the IFR to be homogeneously dispersed in PO matrix, resulting in poor mechanical properties. In response to this problem, the aforementioned three strategies can also be adopted.

However, although some FRs have good performance on flame retardancy, and the obtained PO composites possess acceptable mechanical performance, most of the FRs are still in the stage of laboratory research. New FR materials that can be easily prepared with high efficiency and environmental harmlessness will be the desired ones for flame-retardant PO cable sheath materials in the future.

## Figures and Tables

**Table 2:** Cooperative effect of SiO_2_ as an adjuvant and other FRs.

PO Matrix	SiO_2_ (wt.%) ^a^	Other FRs (wt.%)	Flame Retardancy () ^b^	Mechanical Property () ^b^	Reference
EVA	SiO_2_ (1.5)	layered double hydroxides (LDH) (48.5)	LOI: 30.8% (28.3%)		[63]
EVA	SiO_2_ (5.0)	MH (55.0)	UL-94 (3.0 mm) ^c^: V-0 (V-0) LOI: 39.0% (35%)	σ_t_: 11.1 MPa (10.4 MPa) ε_b_: 70.0% (75.0%)	[64]
EVA	SiO_2_ (2.0)	ATH (53.0)	UL-94 (3.0 mm): No rating, nodripping (No rating, dripping) LOI: 33.2% (35.2%)		[65]
EVA	SiO_2_ (5.0)	ATH (120) + DCP (2.0)	UL-94 (3.0 mm): V-0 LOI: 34.0%	σ_t_: 21.0 MPa ε_b_: 420.0%	[66]
PP	SiO_2_ (1.0)	PCO-900 (3.5) + NOR-116 (1.5)	LOI: 25.7% (25.0%)		[67]

^a^ The concentrations of the additives; ^b^ value for PO composites without SiO_2_ added; ^c^ thickness of the tested sample; σ_t_, tensile strength; ε_b_, elongation at break.

**Table 3:** Examples of MMT/OMMT as adjuvants with conventional FRs in PO composites.

PO Matrix	MMT/OMMT (wt.%)	Other FRs (wt.%)	Flame Retardancy () ^a^	Mechanical Property () ^a^	Reference
LDPE	MMT (2.25)	MH (55.0)	UL-94 (3.0 mm): NC ^b^(NC) LOI: 29.3% (31.9%)		[76]
EVA	MMT (1.0)	ATH (49.0)	UL-94: V-0 LOI: 26.0% (33.0%)	σ_t_: 11.2 MPa (17.4 MPa) ε_b_: 61.0% (21.0%)	[77]
EVA	OMMT (1.0)	ATH (49.0)	UL-94: V-0; LOI: 28.0% (33.0%)	σ_t_: 11.7 MPa (17.4 MPa) ε_b_: 61.0% (21.0%)	[77]
PP	APP-CaMMT (APP:CaMMT = 19:1) (14.3)	Dipentaerythritol (DPER) (5.7)	UL-94 (3.2 mm): V-0 (NC) LOI: 27.5% (21.9%)		[78]
PP	Fe-OMT (4.0)	IFR (APP: PER: melamine polyphosphate (MPP) = 9:4:7) (24.0)	UL-94 (3.0 mm): V-0 (NC) LOI: 30.0% (23.0%)		[79]
PP	Ca-MMT (0.5)	Poly(ethylene glycol) grafted polypropylene (0.5)		σ_t_: 33.5 MPa ε_b_: 132.5%	[80]
LDPE	MMT (4.0) + LDPE grafted maleic anhydride (12.0)	MH (48.0)	LOI: 26.0% (25.0%)	σ_t_: 13.1 MPa (10.8 MPa) ε_b_: 2.8% (1.6%)	[81]
HDPE/ EVA	MMT (5.0)	MH (45.0)	UL-94 (3.0 mm): V-0 (V-0) LOI: 28.3% (28.4%)		[82]
HDPE/ EVA	OMMT (5.0)	MH (45.0)	UL-94 (3.0 mm): V-0 (V-0) LOI: 29.6% (28.4%)		[82]
PP	MMT (1.2)	APP (10.8) + DPER (4.0) + melamine (MEL) (4.0)	UL-94 (3.2 mm): V-0 LOI: 29.8%	σ_t_: 30.0 MPa	[83]
PP	OMMT (2.6)	IFR (28.0) + PP-g-MAH (4.0)	UL-94 (4.0 mm): V-0 (V-0) LOI: 32.8% (30.7%)	σ_t_: 28.1 MPa (30.8 MPa)	[84]
LDPE/ EVA	OMMT (5.0)	ATH (30.0) + MH (15.0)	LOI: 23.3%	σ_t_: 15.6 MPa ε_b_: 33.5%	[85]

^a^ Value for PO composites without MMT/OMMT added. ^b^ Not classifiable.

**Table 4:** Effects of different silicon FRs on the performance of PO matrix.

PO Matrix	FRs Additives (wt.%)	Mode of Action	Results () ^a^	Reference
LDPE	MAPP (28.6) (APP covered with KH-570 (3-(Methylacryl-oxyl) propyltrimethoxy silane) and SiO_2_) + DPER (11.4)	MAPP composites are better dispersed and have good compatibility with the matrix.	UL-94 (10.0 mm): V-0 (V-0); LOI: 28.1% (26.7%); σ_t_: 2.7 MPa (2.4 MPa); ε_b_: 33.8% (24.1%)	[116]
PP	HBPPA-Si (12.5) (hyperbranched polyphosphamide with terminal groups of silane) + APP (12.5)	HBPPA-Si has higher thermal stability and more excellent char formation.	UL-94 (3.2 mm): V-0 (NC); LOI: 27.5% (21.3%); σ_t_: 28.3 MPa (26.3 MPa); ε_b_: 28.0% (24.0%)	[117]
PP	OA-POSS (octa-ammonium-POSS) (1.0) + IFR(APP:PER = 3:1) (19.0)	OA-POSS acts as a plasticizer in the melt.	UL-94 (2.0 mm): V-1 (NC); LOI: 29.7% (24.5%); ε_b_: 24.0% (23.0%)	[118]
PP	TS-POSS (trissulfonic acid propyl-POSS) (1.0) + IFR (APP:PER = 3:1) (19.0)	TS-POSS acts as a plasticizer in the melt.	UL-94 (2.0 mm): V-1 (NC); LOI: 32.4% (24.5%); ε_b_: 27.0% (23.0%)	[118]
PP	APID (polysiloxane containing phosphorus, nitrogen and benzene rings) (10.0) + APP (15.0)	APID acts as blowing agent and carbonization agent.	UL-94 (1.6 mm): V-0 (NC); LOI: 29.8% (24.1%); σ_t_: 31.8 MPa (34.7 MPa); ε_b_: 72.3% (109.9%)	[119]
PP	Si-MCA (6.3) (silicone-containing macromolecular) + APP (18.7)	Si-MCA helps to form a compact and thermostable intumescent char.	UL-94 (3.2 mm): V-0 (NC); LOI: 33.5% (26.0%); σ_t_: 27.4 MPa (25.6 MPa)	[120]
PP	Si-APP (APP modified with polysiloxane) (18.75) + CA (charring agent) (6.25)	Polysiloxane shell can enhance thermal stability.	UL-94 (3.2 mm): V-0 (V-0); LOI: 35.0% (32.7%);	[121]
PP	Polysilsesquioxane (5.0) + IFR (APP:PER = 3:1) (25.0)	The synergism between IFR and polysilsesquioxane enhances char yield and form stable C-Si bonds.	UL-94 (3.0 mm): V-0 (NC); LOI: 36.0% (30.0%); σ_t_: 21.0 MPa (20.5 MPa); ε_b_: 33.0% (39.0%)	[122]
PP	Polysilsesquioxane (5.0) + IFR (APP:PER = 3:1) (30.0)	The synergism between IFR and polysilsesquioxane enhances char yield and form stable C-Si bonds.	UL-94 (3.0 mm): V-0 (V-0); LOI: 39.5% (32.0%); σ_t_: 16.0 MPa (20.0 MPa); ε_b_: 25.0% (32.0%)	[122]
PP	HFR (prepared with γ-Aminopropyltriethoxysilane and other agents) (5.0) + IFR (APP:PER = 3:1) (25.0)	HFR helps to produce more compact intumescent char.	UL-94 (3.0 mm): V-0 (V-0) LOI: 36.0% (32.0%);	[123]

^a^ Value for PO composites without organic silicon added.

**Table 5:** Methods of IFRs modification.

PO Matrix	Methods	IFR Formulation (wt.%)	LOI () ^a^ UL-94 () ^a^	σ_t_ () ^a^ ε_b_ () ^a^	Reference
PP	Modify traditional IFRs with a titanate coupling agent NDZ-201 by ball milling to obtain MIFRs	MIFRs (APP + PER + MEL) (25.0)	31.2% (29.0%) (3.2 mm) ^b^ V-0 (V-2)	29.0 MPa (23.0 MPa) 100.0% (15.0%)	[147]
PP	Use phytic acid (PA) and MF resin to modified APP by supramolecular assembly method to obtain APP@MF-PA	APP@MF-PA (20.0) + CFA (5.0)	35.0% (34.0%) (3.0 mm) V-0 (V-0)		[148]
PP	Decorate the surface of MPP and dialdehyde starch (DAS) by co-microencapsulation technology to obtain M-MPP and M-DAS	M-MPP (15 phr) + M-DAS (15 phr)	28.2% (27.1%) (3.0 mm) V-1 (V-1)		[149]
PP	Microencapsulate APP with HBPE by KH-550 to obtain K-HBPE@APP	K-HBPE@APP (25.0)	34.2% (31.0%) (3.2 mm) V-0 (V-1)	21.0 MPa (24.0 MPa) 375.0% (83.0%)	[140]
PP	Use DPER, 4, 4′-diphenylmethane diisocyanate (MDI) and MEL to microencapsulate APP in situ polymerization to obtain MAPP	MAPP (30.0)	32.1% (22.0%) (3.2 mm) V-0 (NC)		[150]
PP	Modify UF by KH-550 to obtain M-UF	APP (20.0) + M-UF (10.0)	29.5% (22.0%) (3.2 mm) V-0 (NC)	19.4 MPa (17.9 MPa) 11.4% ^c^ (5.6%)	[146]
PP	Introduce DOPO into the molecular structure of APP to obtain DOPO-modified APP	DOPO-modified APP (30.0)	30.1% (24.2%) (1.6 mm) V-0 (NC)	31.6 MPa (29.8 MPa) -	[151]
PP	Microencapsulate APP with MEL, PER, and MDI via in situ two-step surface polymerization to obtain MAPP	MAPP (30.0)	25.0% (20.0%) (3.0 mm) V-1 (NC)		[152]
PP	Microencapsulate APP-II with MF resin via in situ polymerization to obtain MFAPP-II	MFAPP-II (30.0) + PER (8.3)	39.7% (39.0%) (3.0 mm) V-0 (V-0)		[153]
PP	Modify APP-I with ethylenediamine via ion exchange reaction to obtain MAPP	MAPP (40.0)	32.5% (20.9%) (3.2 mm) V-0 (NC)		[154]

^a^ Value for PO composites with unmodified IFR added. ^b^ Thickness of the tested sample. ^c^ The elongation at break of pure PP is 17.8% in Reference [146].

**Table 6:** Studies about cooperative effect of compounds with IFR systems.

PO Matrix	IFRs (wt.%)	Adjuvants (wt.%)	LOI () ^a^ UL-94 () ^a^	σ_t_ () ^a^ ε_b_ () ^a^	Reference
LLDPE	IFRs (ADP@KH-560: neopentyl glycol:MEL = 1.5:1:1) (25.0) ADP@KH-560: aluminum diethylphosphinate modified with KH-560;	ZB (5.0)	28.7% (28.5%); (3.0 mm) ^b^ V-0 (V-0)		[185]
PP	IFRs (APP:DPER = 3:1) (23.5) DPER: double pentaerythritol	Kaol-GLY (1.5) Kaol-GLY: introduced glycine into layers of kaolinite.	32.9% (27.3%); (3.0 mm) V-0 (NC)		[186]
PP	Single-component IFR (APP + PER + MEL) (24.0)	polyhedral oligomeric silsesquioxane (1.0)	31.2% (29.7%); (1.6 mm) V-0 (V-1)	29.0 MPa (26.0 MPa) -	[187]
PP	IFRs (APP:PER = 3:1) (20phr) + PP-g-MAH (4phr)	4ZnO·B_2_O_3_·H_2_O (1 phr)	31.2% (28.9%); (4.0 mm) V-2 (V-2)	- 1084.0% (1146.0%)	[188]
EVA	mixed FR (IFRs (APP:PER:MEL = 3:1:1) + FeOOH) (19.0)	Fumed silica (1.0)	20.8% (21.3%); (3.0 mm) V-2 (V-2)	19.9 MPa (14.2 MPa); 675.0% (615.0%)	[189]
LDPE	IFRs (SiO_2_@MAPP:DPER = 2:1) (23.6)	KU (1.4) KU: The intercalation of modified kaolin with urea.	27.2% (24.1%); (3.0 mm) V-1 (NC)	16.6 MPa (16.1 MPa); 554.0% (512.0%)	[190]
PP	IFRs (APP:PER = 2:1) (13.5)	PAMA-Mn (4.5) PAMA-Mn: MEL phytate supramolecular nanosheet FR incorporating manganese ion	31.8% (26.5%); (3.2 mm) V-0 (NC)		[191]
PP	IFRs (MCAPP:PEPA = 2:1) (25.0) MCAPP: APP microencapsulated with MEL	P-type hydrated silica aluminate (HSA-P) (1.5)	35.1% (31.2%); (3.0 mm) V-0 (V-2)		[192]
PP	IFRs (MCAPP:PEPA = 2:1) (25.0) MCAPP: APP microencapsulated with MEL	La-loaded for P-type hydrated silica aluminate(HSA-P-La) (1.5)	37.5% (31.2%); (3.0 mm) V-0 (V-2)		[192]
PE	IFRs (APP:PER = 3:1) (25.6)	Yb(OTf)_3_ (0.4)	25.9% (24.2%); V-0 (NC)		[193]
PP	IFRs (APP:PER = 3:1) (19.0)	Co-MMT (MMT intercalation cobalt compounds) (4.0)	32.1% (26.5%); (3.2 mm) V-0 (V-2)		[194]
PP	IFRs (APP:PER = 3:1) (25.0)	scCO_2_ (7.0)	35.8% (32.8%); (3.2 mm) V-0 (V-2)		[195]
PP	IFRs (OS-MCAPP:CFA = 3:1) (29.7) OS-MCAPP: silica-gel microencapsulated ammonium polyphosphate	NiPO-NT (0.3) NiPO-NT: nickel phosphate nanotubes	33.9% (29.8%); (3.0 mm) V-0 (V-0)		[196]
PP	IFRs (APP:PER = 2:1) (25.0)	Ni (4.0)	34.2% (29.0%); (3.0 mm) V-0 (V-1)		[197]
PP	IFRs (APP:PER = 2:1) (25.0)	Ni-Al (Ni:Al = 9:1) (4.0)	36.8% (29.0%); (3.0 mm) V-0 (V-1)		[197]
PP	IFRs (APP:PER = 2:1) (25.0)	Ni-Mg (Ni:Mg = 9:1) (2.0)	38.1% (29.0%); (3.0 mm) V-0 (V-1)		[197]
PP	IFRs (APP:PER = 2:1) (25.0)	Ni-Cu (Ni:Cu = 9:1) (4.0)	36.6% (29.0%); (3.0 mm) V-0 (V-1)		[197]

^a^ Value for PO/IFR composites without adjuvants. ^b^ Thickness of the tested sample.

**Table 7:** Studies about the new molecules or their complexes employed as acid sources or carbon agents.

PO Matrix	New IFR (wt.%)	Molecular Structure or Synthetic Method of the Acid/Carbon Sources	LOI UL-94	σ_t_ ε_b_	Reference
PP	Acid source: APP (16.7) Carbon source: BTETP (8.3)	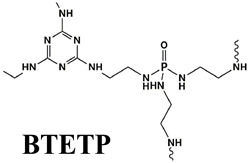	32.3% V-0		[207]
PP	Acid source: APP modified with piperazine (18.75) Carbon source: ATPIP (6.25)	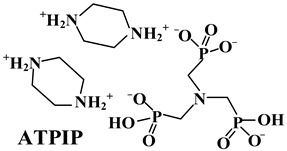	30.0% V-0	35.5 MPa 40.3%	[208]
PP	Acid source: APP (12.5) Carbon source: HBPPA-Si (12.5)	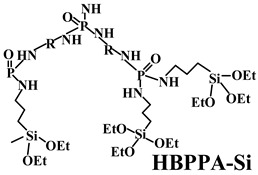	27.5% V-0	28.3 MPa 28.0%	[117]
PP	Acid source: PPA-C (15.0) Carbon source: PER (5.0)	PPA-C was prepared with pyrophosphoric acid (PPA) and cytosine (C) via a one-pot method.	30.0% V-0		[209]
PP	Acid source: GO-APP (22.5) Carbon source: PER (7.5) Adjuvants: maleic anhydride-grafted polypropylene (1.0); antioxygen 1010 (1.0)	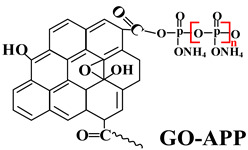	31.2% V-0	25.0 MPa 47.0%	[210]
PP	Acid source: silica-gel-microencapsulated APP (20.0) Carbon source: PEIC (10.0)	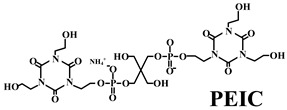	32.7% V-0	15.3 MPa 11.2% ^a^	[211]
PP	Acid source: APP (5.0) Carbon source: HPPU (20.0)	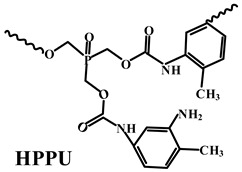	27.0% V-0		[212]
PP	Acid source: APP (20.0) Carbon source: SCTCFA-ZnO (10.0)	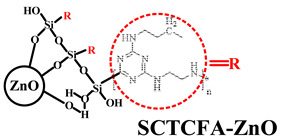	36.2% V-0		[213]
PP	Acid source: APP (18.0) Carbon source: NFR (12.0)	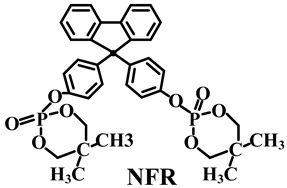	31.0% V-0	66.5 MPa 19.7%	[214]
PP	Acid source: APP (15.0) Carbon source: PEPAPC (5.0)	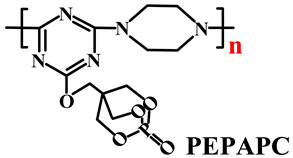	28.0% V-0		[215]
PP	Acid source: APP (18.75) Carbon source: HBPPDA (6.25)	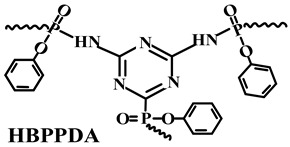	30.6% V-0		[216]
PP	Acid source: APP (22.5) Carbon source: CNCD-DA (7.5)	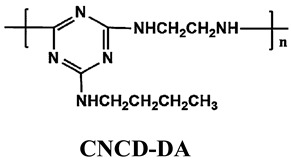	36.5% V-0		[217]
PP	Acid source: APP (18.24) Carbon source: MTEC (4.56) Adjuvant: SiO_2_ (1.2)	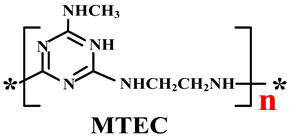	30.7% V-0		[218]
PP	Acid source: APP (10.0) Carbon source: PN-HBP (10.0)	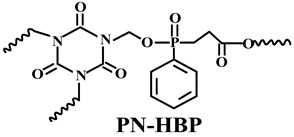	30.0% V-0		[219]
PP	Acid source: IMAPP (17.2) Carbon source: DPER (7.8) Adjuvants: 1. antioxidant 1010 (0.1); 2. antioxidant 168 (0.2)	IMAPP is prepared by the chemical reaction between aluminum chloride and ammonia	32.1% V-0		[220]
LDPE	Acid source: APP (20.0) Carbon source: CNCA-DA (10.0)	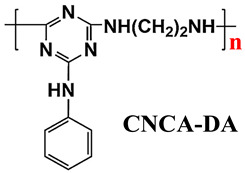	30.9% V-0		[221]
EVA	Acid source: APP (18.0) Carbon source: CNCO-HA (12.0)	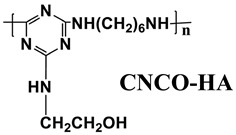	30.9% V-0		[222]
PP	Acid source: APP (12.5) Carbon source: CNCO-HA (12.5)	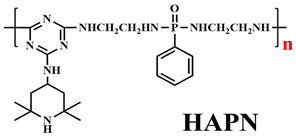	29.5% V-0		[223]
PP	Acid source: APP (18.75) Carbon source: TBMC (6.25)	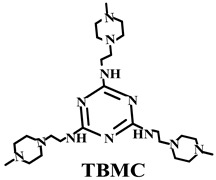	30.5% V-0	21.2 MPa 36.9%	[224]

^a^ The elongation at break of pure PP is 11.3% in Reference [211].

**Table 8:** Studies on the cooperative flame retardation of EG.

PO Matrix	EG FRs (wt.%)	Other Additives (wt.%)	LOI () ^a^ UL-94 () ^a^	σ_t_ () ^a^ ε_b_ () ^a^	Reference
HDPE/ EVA	MEG (modified by DOPO) (5.0)	MH/ATH (3/2) (45.0)	38.4% (29.0%); (4.0 mm) V-0 (NC)	21.5 MPa (20.3 MPa); 13.6% (50.8%)	[235]
HDPE/ EVA	MEG (modified by DOPO) (4.0)	1.MH/ATH (3/2) (45.0); 2.zinc borate (1.0)	37.1% (29.4%); (4.0 mm) V-0 (NC)	23.0 MPa (20.8 MPa); 15.7% (54.8%)	[235]
LDPE	EG (5.0)	1.RP_PMHS_ (modified with poly(methylhydrosiloxane)) (5.25); 2.ATH_Mgst_ (modified with magnesium stearate) (5.25)	25.4% (22.6%); (2.2 mm) V-0 (V-2)	9.3 MPa (9.3 MPa); 64.9% (112.7%)	[236]
LLDPE/EVA	MEG (modified with DOPO and silane coupling agent) (10.0)	MH/ATH(3/2) (40.0)	32.7% (29.6%); (2.7 mm) V-0 (V-2)		[237]
LLDPE/EVA	MEG (modified with DOPO and KH560) (4.0)	1.MH/ATH (45.0); 2.zinc borate (1.0)	31.7% (29.0%); (4.0 mm) V-0 (NC)		[238]
PP	MEG (modified with DOPO and silane coupling agent) (30.0)		25.3% (18.1%); (2.7 mm) V-0 (NC)	27.5 MPa (31.0 MPa); 9.1% (446.3%)	[239]
EVA	EG (27.0)	palygorskite@boric acid@dodecylamine (PGS@B-N) (3.0)	37.7% (21.2%); (3.0 mm) V-0 (NC)	13.0 MPa; 1007.3%	[240]
EVA	EG (10.0)	LDH (20.0)	29.7% (27.0%); (3.0 mm) V-0 (NC)		[241]
EVA	EG (20.0)	APP (10.0)	30.7% (20.3%); (3.0 mm) V-0 (V-2)		[242]
HDPE/ EVA	EG (20.0)			10.1 MPa (15.6 MPa); 315.5% (517.5%)	[243]

^a^ Value for PO and other additives without EG.

**Table 9:** The most effective FRs for PP, EVA, and LDPE, respectively.

PO Matrix	LOI	UL-94	σ_t_	ε_b_	Flame Retardants (wt.%)	Reference
PP	31.4%	V-0	35.0 MPa	132.0%	IFR (APP/PER = 3/1) (20) + nano-CB (5) + POE-MA (8)	[173]
	34.2%	V-0	21.0 MPa	375.0%	K-HBPE@APP (use HBPE to microencapsulate APP via KH-550 to obtain K-HBPE@APP) (25)	[140]
	34.3%	V-0	32.0 MPa	200.0%	MIFRs (modify traditional IFRs with a titanate coupling agent NDZ-201 by ball milling to obtain MIFRs) (25) + APID (5)	[147]
EVA	34.0%	V-0	21.0 MPa	420.0%	SiO_2_ (5.0) + ATH (120) + DCP (2)	[66]
	30.5%	V-0	12.0 MPa	732.0%	EG (10) + AHP (5)	[234]
	37.7%	V-0	13.0 MPa	1007.3%	EG (27) + palygorskite@boric acid@dodecylamine(PGS@B-N) (3.0)	[242]
LDPE	28.1%	V-0	2.6 MPa	33.8%	MAPP (28.6) + DPER (11.4)	[116]
	27.2%	V-1	16.6 MPa	554.0%	IFRs (SiO_2_@MAPP:DPER = 2:1) (23.6) + KU(the intercalation of modified kaolin with urea) (1.4)	[190]
	25.4%	V-0	8.3 MPa	103.8%	EG (5) + RPPMHS (modified with poly(methylhydrosiloxane)) (5.25) + ATHMgst(modified with magnesium stearate) (5.25) + POE (4)	[238]

## Data Availability

No new data were created or analyzed in this study.
